# Implementation of clerkship-specific spaces for debriefing at Vanderbilt University School of Medicine

**DOI:** 10.1080/10872981.2026.2630522

**Published:** 2026-02-17

**Authors:** Nina Curkovic, Logan Locascio, Sachin Aggarwal, Luke Finck, Jessica Turnbull, Maie El-Sourady, Travis W. Crook

**Affiliations:** a Vanderbilt University School of Medicine, Nashville, TN, USA; b University of Texas Southwestern Medical Center, Department of Dermatology, Dallas, TX, USA; c University of Texas Southwestern Medical Center, Department of Internal Medicine, Dallas, TX, USA; d Vanderbilt University Medical Center, Center for Biomedical Ethics and Society, Nashville, TN, USA; e Vanderbilt University Medical Center, Department of Internal Medicine, Section of Palliative Care, Nashville, TN, USA; f Vanderbilt University Medical Center Department of Pediatrics, Nashville, TN, USA; g The University of Texas at Austin Dell Medical School, Austin, TX, USA

**Keywords:** Debrief, undergraduate medical education, medical students, resilience, clerkship: clinical, moral distress, curriculum, wellness

## Abstract

Difficult experiences like challenging clinical situations, patient death, and navigation of team dynamics during clerkships can lead to distress for medical students during the transition to the clinical learning environment. Evidence suggests that debriefing and reflecting on distressing experiences benefit medical students; however, barriers to doing so persist. A needs assessment at Vanderbilt University School of Medicine (VUSM) demonstrated that additional opportunities for real-time, clerkship-specific debriefing were desired by second-year clerkship medical students. The CIRCLE (Cultivating Increased Resilience in the Clinical Learning Environment) Lunch & Debrief initiative was created to address this need. CIRCLE sessions were conducted once per 8-week clerkship block for two cohorts of VUSM second-year clerkship students. Students were optionally surveyed before and after sessions from December 2022 through May 2024. Student resilience was assessed using the Connor Davidson Resilience Scale© (CD-RISC 10). The proportion of students reporting distress stemming from health care team interactions and patient death demonstrated significant increases from blocks 1–3 to blocks 4–5. More students reported participation in debriefing following a distressing experience with peers than with faculty across all pre-session surveys (78% vs 37%; *p* = 1.6e-10). Reported comfort with asking to debrief with peers increased from 80% to 94% before and after sessions (*p* = 0.006) and with faculty from 52% to 73% (*p* = 0.004.) Of post-session survey respondents (*n* = 71), 96% indicated they would attend a similar session in the future. Perceived support by the medical school curriculum to navigate emotional distress as a clerkship student increased from 69% to 90% when comparing pre- and post-session survey responses, respectively (*p* = 0.003). Overall, reception of the CIRCLE Lunch & Debrief initiative by students was positive and was associated with increased perceived institutional support and student comfort with asking to debrief with both faculty and peers.

## Introduction

Clerkships mark a pivotal transition point in medical student education, and difficult clinical encounters, team dynamics, and uncertainty during this time may lead to distress for medical students. Vanderbilt University School of Medicine and many other medical schools have shifted towards accelerated preclinical curricula [1]. This early exposure to the clinical learning environment exposes students to emotionally and/or morally distressing clinical experiences sooner in their professional development than ever before [2,3].

Reflection and debriefing have been shown to be cathartic and contribute to increases in medical student empathy and resilience scores [4–6]. However, commonly cited barriers to debriefing in the clinical environment include time constraints and cultural constraints, wherein students may perceive disinterest, misunderstanding, or criticism from their team members [2,3,7]. Consequently, medical students are more likely to seek debriefing from peers, though debriefing with experienced team members, such as attending physicians, has been positively received when facilitated [3–5].

An internal needs assessment at Vanderbilt University School of Medicine (VUSM) showed that existing curricular opportunities for debriefing and reflection lacked specificity and real-time applicability to variable student experiences on different clerkships. Thus, an initiative for clerkship-specific spaces for debriefing, known as CIRCLE (Cultivating Increased Resilience in the Clinical Learning Environment) Lunch & Debrief, was developed.

We hypothesised that students who participated in CIRCLE would report increased comfort with debriefing as well as increased levels of resilience by the end of the year when compared to the beginning. Herein, we present data collected regarding student reception, subsequent comfort with debriefing, and resilience from the first two years of this newly implemented initiative at Vanderbilt University School of Medicine.

## Materials and methods

### Setting and study design

VUSM clerkship students rotate through 6 clerkships during the second year of medical school in five 8-week blocks: Internal Medicine (8 weeks), Pediatrics (8 weeks), Surgery (8 weeks), Obstetrics and Gynecology (8 weeks), Neurology (4 weeks), and Psychiatry (4 weeks). CIRCLE Lunch & Debrief is an optional once-per-clerkship, lunchtime (12–1 pm) session for debriefing occurring on the 5th Friday of each 8-week clerkship block. Sessions are specific to each clerkship with VUSM faculty members serving as facilitators from their respective specialties.

Prior to the advent of the programme, a curriculum committee comprising 6 students at the Vanderbilt University School of Medicine selected the facilitators. The committee was instructed to make recommendations regarding suitable candidates for attending physicians in specialties representing the clerkship blocks. Because neurology and psychiatry clerkships are 4 weeks in length, facilitators for the neurology and psychiatry cohorts include faculty from both specialties based on early feedback from students. Considerations for selection included history with medical education efforts and positive reputation with students.

Upon selection, and at the start of subsequent academic years, faculty facilitators were invited to attend a one-hour workshop led by the CIRCLE directors. In this workshop, the directors and faculty facilitators discussed the history of debriefing and context of debriefing in the medical school environment as well as the recommended schedule for each hour-long session. Each session consisted of group introductions; debriefing; sharing of coping skills, strategies, and resources; and additional time for remaining questions or comments as well as to allow for completion of the post-session survey. The debriefing components were intended to be open-ended without predetermined conversation topics. Faculty facilitators were recognised with a ‘Trusted Debriefer’ pin. Following the first CIRCLE session, faculty were encouraged to wear their pin in all clinical settings to promote organic student debriefing in the clinical setting. Facilitators also received supplementary reading related to medical education efforts.

We surveyed two cohorts of VUSM second-year clerkship students before and after CIRCLE sessions from December 2022 through May 2024. Students were informed that CIRCLE sessions were debriefing discussions among faculty members and students regarding difficult encounters on their specific clerkship, and attendance at sessions was optional. Pre-session and post-session surveys were made electronically available on REDCap® (Research Electronic Data Capture) to all clerkship students via email 1–3 days prior to each session and immediately after each session [8,9]. Participation in surveys was voluntary and anonymous. We additionally surveyed one cohort of third-year, post-clerkship phase medical students in December 2022 who had not participated in CIRCLE sessions.

Surveys included several questions aimed at assessing sources of distress from clerkship experiences, experience and comfort with peer and faculty debriefing, and feedback on the initiative’s structure, impact, and reception. Student resilience was assessed using the Connor Davidson Resilience Scale© (CD-RISC 10) at the first and last session of the academic year for each cohort [10]. Third-year, post-clerkship phase students who did not receive CIRCLE sessions during clerkships also completed the CD-RISC 10 upon commencement of the first CIRCLE session. All collected information was de-identified.

This study was granted an exemption from the Institutional Review Board at Vanderbilt University. Students were surveyed with permission and oversight from Vanderbilt University School of Medicine administration.

### Statistical analysis

Descriptive and statistical analysis was conducted using R (version 4.2.2) [11]. Continuous variables were compared using the Mann-Whitney Wilcoxon test. Categorical variables were tested between groups using the Fisher exact test. Statistical tests were conducted with a significance level of 0.05.

## Results

From December 2022 to May 2024, 9 debriefing sessions were delivered to 2 cohorts of second-year clerkship medical students, each consisting of approximately 100 students. A total of 433 attendees participated in the debriefing sessions across all nine sessions (mean: 48 attendees amongst all clerkships per session). One to three facilitators were present at a time for a given block across the study period. The number of students in attendance per group ranged from 2–17. A total of 120 pre-survey responses and 71 post-survey responses were collected.

### Student experiences with sources of distress

**Figure 1.**
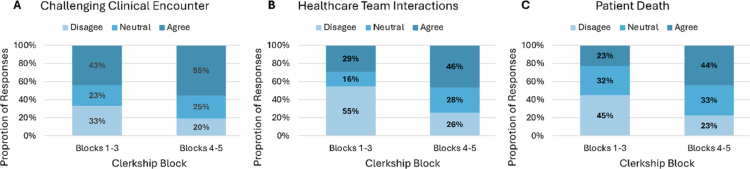
Student responses regarding the experience of distress from A) challenging clinical encounters B) interactions among a health care team or C) involvement in the care of a patient who died from pre-session surveys across clerkship blocks. Students reporting distress from health care team interactions increased from 29% vs 46% from blocks 1–3 to blocks 4–5; *p* = 0.006. Distress from patient death also increased from 23% to 44% (blocks 1–3 vs. blocks 4–5; *p* = 0.033). Change in student distress related to a challenging clinical encounter was not significant (blocks 1–3 vs. blocks 4–5; *p* = 0.268).

Student reports of distress related to interactions among the healthcare team and involvement in the care of a patient who died significantly changed between clerkship blocks 1–3 vs. blocks 4–5 in the following parameters. There was a statistically significant difference in students’ experiences of distress from health care team interactions (*p* = 0.006). Students agreeing with having experienced distress from health care team interactions increased from 29% vs 46% from blocks 1–3 to blocks 4–5. Student reported involvement in the care of a patient who died increased as students progressed through clerkships from 67% to 90% (blocks 1–3 vs. blocks 4–5; *p* = 0.013). Distress from patient death also increased from 23% to 44% (blocks 1–3 vs. blocks 4–5; *p* = 0.033). Student distress related to a challenging clinical encounter similarly appeared to increase in later clerkship blocks, though this change was not significant (blocks 1–3 vs. blocks 4–5; *p* = 0.268) (Figure 1).

### Student experiences and comfort with debriefing

**Figure 2.**
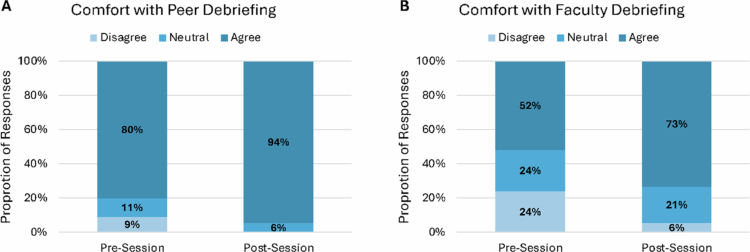
Comparison of student responses of reported comfort with asking to debrief with A) peers or B) faculty on pre- and post-session survey responses. Reported comfort with asking to debrief with peers increased from 80% to 94% before and after sessions (*p* = 0.006) and with faculty from 52% to 73% (*p* = 0.004).

More students reported participation in debriefing following a distressing experience with peers than with faculty across all pre-session surveys (78% vs 37%; *p* = 1.6e-10). Of students reporting prior debriefing with faculty (*n* = 44), 95% reported that they found the debriefing helpful. Comparison of pre- and post-session survey responses demonstrated an increase in comfort with asking to debrief with both peers and faculty. Comfort with asking to debrief with peers increased from 80% to 94% before and after sessions (*p* = 0.006) and with faculty from 52% to 73% (*p* = 0.004) (Figure 2).

### Student reception of sessions

Sixty-nine percent of students in pre-session surveys indicated they felt supported by the medical school curriculum to navigate emotional distress as a clerkship student, compared to 90% of students in post-session surveys (*p* = 0.003). Among post-session survey responses (*n* = 71), 96% of participants agreed or strongly agreed they would attend a similar session in the future. Ninety-three percent of participants agreed or strongly agreed that the presence of faculty facilitators was helpful, whereas most students disagreed (48%), strongly disagreed (1%), or were neutral (23%) about senior medical students participating in future sessions. Themes in students’ written comments about motivation for attending the sessions included value in hearing classmates’ thoughts and experiences on clerkships, value in advice from faculty, a space for vulnerability and desire for processing recent clerkship experiences, and the opportunity to be in an enjoyable space with peers for lunch. Selected comments grouped by theme are shown in Table 1.

**Table 1.** Selected comments from student responses to the post-survey question ‘*Why did you attend this session?’* grouped by common themes.

Theme	Selected comments
Prior positive experience and catharsis	*I attended this session to sit down with my colleagues and mentors, eat lunch, and chat about my experiences on clerkships so far. It really is humanising and therapeutic to have a small group of students sharing encounters in a safe space.*
	*I really benefitted and enjoyed the paediatrics one the first block and I think its important that we normalise these conversations*
	*Because I love the conversations I got to have last time. I think it addresses the important things in medicine that are often not mentioned*
	*I look forward to this every block! Such an important space to debrief thoughts/feelings that come up during clerkship*
	*I attended the session because I find it cathartic to discuss ethical issues and emotions felt on service. I enjoy hearing my classmates' thoughts and sharing mine. Also, free lunch!*
Understanding and supporting peers	*To hear about my classmates' experiences and debrief*
	*Wanted to see what issues people were thinking of and coming to terms with*
	*To understand the experiences of other students in IM, particularly with regard to end of life conversations.*
	*To support classmates, discuss some things on my mind, and have lunch*
	*Enjoy processing with and listening to others' stories.*
	*To see what experiences my classmates have been having on the clerkship and become more aware of things that aren't so good*
Advice from faculty	*To hear others' perspectives on difficult topics, receive guidance from attendings*
	*I was conflicted about some of the things I had seen, and feelings I had about paediatric Psychiatry that I wanted to discuss with some of the attendings/upper levels to see how they address those emotions.*
	*To debrief with faculty and see what others have experienced*
	*To share and hear classmates' perspectives on the challenges and triumphs of the clerkship. And have attendings guide the conversation and ask thought-provoking questions that causes us to reflect.*
Desire to debrief	*I wanted a chance to debrief on rough/difficult encounters.*
	*To listen to other people's experiences and be able to share difficult things I encountered*
	*I wanted to debrief about difficult encounters!*
	*I think these conversations are good to be normalised and to create a space for them*
	*To reflect on my experiences from the past 5 weeks.*
Space for discussion and food	*Break from clerkships and time to talk things through with people.*
	*It's a nice forum to just talk*
	*A space to talk about things (also free food)!*
	*Free lunch; opportunity to debrief*

### Resilience scores

Mean CD-RISC scores across the two clerkship cohorts participating in CIRCLE sessions were 27.2 (±8.4) at the start of the academic year (*n* = 31) vs 28.3 (±6.9) at the end of the academic year (*n* = 31) (*p* = 0.783). The mean CD-RISC of a cohort of third year medical students administered concomitantly with the inaugural CIRCLE session was 30.1 (*n* = 16). There was no statistical significance in CD-RISC score comparisons among groups.

## Discussion

Although clerkships represent a period of significant learning, they can also be a source of distress for students, as reflected in our study cohort’s survey responses throughout the academic year. The majority of surveyed students reported either agreement or neutrality regarding experiences of distress stemming from challenging clinical encounters, interactions among a health care team, and involvement in the care of a patient who died as the academic year progressed. Interestingly, despite student reports of experiences with patient death increasing in frequency throughout the clerkship year, students reported higher rates of distress related to patient death later in the clerkship year. These findings highlight the need for targeted interventions to address distressing experiences during clerkships.

Survey responses assessing attitudes and experiences regarding debriefing among students participating in the CIRCLE initiative aligned with trends reported in prior literature [3–5]. Students reported most frequently debriefing with peers, but when debriefing with senior team members, such as attending physicians, they reported positive experiences. Notably, pre- and post-session survey comparisons demonstrated a significant increase in student comfort with initiating debriefing discussions with both peers and faculty. However, no significant differences in resilience, as measured by the CD-RISC, were seen among groups likely due to small sample size. Despite this, perceived institutional support to navigate emotional distress as a clerkship student significantly increased.

Although the conclusions drawn herein are limited by small sample size and by the voluntary nature of the survey data collection, CIRCLE sessions were well attended—with approximately half of all clerkship students participating, on average—and received positive feedback from both students and participating faculty. Faculty facilitators were selected based on student recommendations, underwent faculty development, and were recognised with a ‘Trusted Debriefer’ pin. While not formally evaluated, these pins were designed to serve as a signal to promote organic debriefing outside of these sessions, addressing existing cultural constraints cited as barriers to debriefing.

The CIRCLE Lunch & Debrief initiative represents one approach to supporting medical students in navigating the emotional challenges inherent in the clinical learning environment. By sharing our experience with this initiative, we aim to foster cultural change surrounding emotional debriefing and inspire the development of additional programmes to better equip future physicians to thrive in the evolving landscape of medical practice.

## Data Availability

Data is available upon reasonable request.
